# Clinical Trials with Pegylated Liposomal Doxorubicin in the Treatment of Ovarian Cancer

**DOI:** 10.1155/2013/898146

**Published:** 2013-03-14

**Authors:** Carmela Pisano, Sabrina Chiara Cecere, Marilena Di Napoli, Carla Cavaliere, Rosa Tambaro, Gaetano Facchini, Cono Scaffa, Simona Losito, Antonio Pizzolorusso, Sandro Pignata

**Affiliations:** Department of Urology and Gynecology, National Cancer Institute, 80131 Naples, Italy

## Abstract

Among the pharmaceutical options available for treatment of ovarian cancer, increasing attention has been progressively focused on pegylated liposomal doxorubicin (PLD), whose unique formulation prolongs the persistence of the drug in the circulation and potentiates intratumor accumulation. Pegylated liposomal doxorubicin (PLD) has become a major component in the routine management of epithelial ovarian cancer. In 1999 it was first approved for platinum-refractory ovarian cancer and then received full approval for platinum-sensitive recurrent disease in 2005. PLD remains an important therapeutic tool in the management of recurrent ovarian cancer in 2012. Recent interest in PLD/carboplatin combination therapy has been the object of phase III trials in platinum-sensitive and chemonaïve ovarian cancer patients reporting response rates, progressive-free survival, and overall survival similar to other platinum-based combinations, but with a more favorable toxicity profile and convenient dosing schedule. This paper summarizes data clarifying the role of pegylated liposomal doxorubicin (PLD) in ovarian cancer, as well as researches focusing on adding novel targeted drugs to this cytotoxic agent.

## 1. Introduction

Ovarian cancer (OvCa) is the leading cause of death from gynaecological malignancies with an estimated 65697 new cases and 41448 deaths every year in Europe [1]. Approximately 15% of women present with disease localized in the ovaries and in this group surgery allows a 5-year survival in more than 90% of the cases. However, the majority of women present at the diagnosis with advanced disease (International Federation of Gynaecological Oncology (FIGO) stage III-IV) and their survival at 5 years is poor, currently less than 30% [2]. 

The main reasons for the high mortality rate are the lack of symptoms accompanying this tumor, in addition to the lack of an effective screening strategy for the overall population, and, lastly, the limited results obtained with standard medical treatments. 

The standard of care for the management of OvCa patients includes surgery for staging and optimal cytoreduction (no residual tumour) followed by a platinum/taxane chemotherapy combination [3, 4]. Recently bevacizumab has been approved in stage IIIb-IV cancer in combination and as a single-agent maintenance after carboplatin-paclitaxel [5, 6]. Although chemotherapy obtains high objective response rates even in patients with an advanced tumor stage, the vast majority of patients will experience tumor progression and require further therapy [7, 8].

Many strategies have been implemented in order to improve these unsatisfactory results and new drugs have been investigated.

In this context, among the pharmaceutical options currently available for medical treatment of ovarian cancer (OvCa), greater emphasis has been placed progressively on pegylated liposomal doxorubicin (PLD) (Doxil in the USA; Caelyx in Canada and Europe), which was approved in 1999 by the FDA and in 2000 by the European Medicines Evaluation Agency (EMA) as single agent for treatment of advanced OvCa patients failing first-line platinum-based treatment. Moreover, phase III trials have been already conducted and results suggest further role for PLD in salvage setting and in front-line treatment in combination with other therapeutic drugs. The aim of this paper is to summarize data showing the role of pegylated liposomal doxorubicin (PLD) in the management of epithelial ovarian cancer.

## 2. Pegylated Liposomal Doxorubicin (PLD): Development, Structure and Pharmacokinetic Features

Anthracyclines have been for years among the drugs administered for the majority of gynecologic cancers. Before taxanes were introduced into first-line therapy of ovarian cancer, anthracyclines demonstrated a comparable efficacy, in monochemotherapy, with alkylating agents and superiority of the combination of both when compared to single-agent therapy. Furthermore, meta-analysis data suggest that the addition of anthracyclines to cisplatin might be advantageous compared to using cisplatin alone [9, 10].

Attempts have been made to introduce anthracyclines in combination with carboplatin-paclitaxel. In the randomized trial, conducted by the AGO group in collaboration with the French group GINECO, the addition of epirubicin (TEC arm) to the platinum/paclitaxel (TC arm) combination in first-line ovarian cancer treatment patients showed a not statistically significant advantage of about 5 months in median overall survival time (45.8 versus 41.0 months, HR 0.93) [11], with no progression-free survival benefit (18.4 versus 17.9 months, HR 0.95) at the price of a greater toxicity of TEC versus TC arm (grade 3/4 hematologic, nausea/emesis, mucositis, and infections). Despite the antitumor activity in ovarian cancer, the clinical use of conventional anthracyclines is limited by their associated side effects. The haematological toxicity and the cumulative and irreversible cardiac damage (congestive heart failure) are the more common side effects, dose limiting, of anthracyclines. As far as it is elucidated, cardiotoxic events take place by increasing oxidative stress, suppression of gene expression, and induction of apoptosis on cardiac tissue [12] with clinical manifestations reaching from acute cardiac heart failure to chronic cardiac insufficiency. Several treatment strategies, including the development of new formulations for delivering the cytotoxic agents (as liposomes encapsulation), have been proposed to improve the therapeutic index of anthracyclines [13]. The inclusion of anthracyclines in a liposomal structure has been proposed to reduce side effects and to enhance the antitumor activity. In this paper, we will focus on the pharmacologic properties of pegylated liposomal doxorubicin (PLD), a new available formulation of doxorubicin that is encapsulated in a pegylated liposome [14, 15]. The size of the liposomes, approximately 100 nm, prevents them from entering tissues with tight capillary junctions, such as the heart and gastrointestinal tract [16]. In contrast to other nanoparticles, the liposomal shell is surrounded by a polyethylene glycol (PEG) layer which represents a hydrophilic protective barrier between the liposome and the microenvironment, thus preventing the activation of the reticuloendothelial system, that leads to the destruction of the liposomal structure and release of the free drug. Liposomal drug delivery to cancer cells can occur in vivo by two different pathways: passive and active targeting. In contrast to normal vessels, the vessels of the tumor are tortuous, dilated, have morphologically abnormal endothelial cells, and are leaky due to large spaces between pericytes [17]. These physical characteristics allow more extravasation of the liposomes into the tumor, with higher cell concentration of the drug. The lack of functional lymphatic drainage in tumours prevents the outflow of extravasated liposomes, allowing doxorubicin accumulation in the tumour extracellular fluid. These liposomes will gradually release the entrapped drug in the vicinity of tumour cells, thus increasing the tumour-drug exposure [18]. This mechanism of passive targeting is known as “enhanced permeability and retention (EPR) effect” [19]. 

The efficacy and safety of PLD has been evaluated in a variety of different tumor models, including several human xenograft models supporting its introduction in cancer treatment [15]. In every model examined, PLD was more effective than the same dose of free doxorubicin in inhibiting or halting tumor growth, in preventing metastasis, and/or in prolonging survival of the tumor-bearing animals [20, 21]. The pharmacokinetic and tissue distribution studies in these models suggest that the greater persistence, particularly in tumor tissue, achieved with PLD compared with conventional doxorubicin offers a therapeutic advantage. PLD has well-known pharmacokinetic features, such as long circulation time, minimal (<5%) drug leakage from circulating liposomes, and half-lives of approximately 60–90 h for doses in the range of 35–70 mg/m^2^ in patients with solid tumors [21]. This translates into a PLD AUC approximately 250–1000-fold higher than that of the free drug in humans [22]. PLD pharmacokinetics is best modeled as a one-compartment model displaying linear pharmacokinetics with C-max increasing proportionally with dose [23]. It has also been described as a two-compartment model with an initial half-life of several hours, followed by a more prolonged terminal decline with a half-life of 2-3 days, accounting for the majority of the AUC [22, 24]. After PLD administration, nearly 100% of the drug in the plasma is in the encapsulated form. Moreover, compared to free doxorubicin, PLD plasma clearance is dramatically slower, and its volume of distribution is very small and roughly equivalent to the intravascular volume [22, 24].

These properties, which represent the rational basis for the exploitation of nanoparticle technology, represent the major advantages of PLD compared to conventional doxorubicin in safety profile (lower cardiotoxicity and gastrointestinal toxicity compared to the free drug) [20–25]. 

Based on the previous evidences regarding the role of anthracyclines and the modified toxicity profile of PLD, this agent has been a rational choice for further evaluation as a single-agent and in combination with platinum agents in the treatment of ovarian cancer. 

## 3. Pegylated Liposomal Doxorubicin: Activity in Ovarian Cancer

### 3.1. Phase II Studies with PLD as a Single-Agent or in Combination

The initial studies evaluating PLD have been conducted in recurrent ovarian cancer, as a single-agent monotherapy or in combination with platinum (carboplatin) and later on with trabectedin or other new drugs. 

A summary of phase II studies using PLD as a single agent or in combination regimens in ovarian cancer is presented in Table 1 [26–35].

Nonrandomized phase II trials of PLD in platinum-resistant ovarian cancer patients documented the biological activity of this agent in this clinical setting, with objective response rates of approximately 10–20% being reported in several trials [18, 25, 31]. Data indicated that palmar-plantar erythrodysesthesia (PPE; hand-foot syndrome, toxic acral erythema) and mucositis were the most common toxicities of PLD, reported in up to 50% of treated patients. PPE usually occurs after two or more courses of treatment and the risk of incidence increases with multiple repeated treatments. PPE is related to dose intensity and dose interval rather than to peak dose level. Although not life threatening, PPE can negatively impact the quality of life, and it is a major cause of both dose reduction and treatment discontinuation [61, 62]. As regards the cardiac toxicity, in several trials PLD formulation has been related to a better safety profile compared to conventional doxorubicin [63]. Compared to the 7.5% incidence of irreversible cardiotoxicity at cumulative doses of 400–550 mg/m^2^ reported with doxorubicin [64], most of the studies of PLD showed a lower incidence of cardiac failure even at doses higher than 500 mg/m^2^ [65, 66]. In a prospective trial performed on patients with advanced gynecological malignancies treated with PLD, the cardiac safety was further assessed at histology (endomyocardial biopsies), showing no myocardial damage in patients treated with PLD (median PLD dose of 708 mg/m^2^) [67]. Thus, the optimal cardiac safety profile of PLD may allow a prolonged treatment; encouraging results from a phase II trial in AIDS-related Kaposi's sarcoma patients treated with PLD up to a 2360 mg/m^2^ cumulative dose have been reported [68]. In metastatic breast cancer patients also doses greater than 450 mg/m^2^ were not associated with a significant decrease in LVEF from baseline compared to conventional doxorubicin [69]. In relapsed ovarian cancer patient responding to second-line chemotherapy, a maintenance therapy with PLD for more than 1 year has been reported to be safe by Andreopoulou et al., with no cardiac event reported [70].

Different schedules and doses have been investigated in an effort to improve tolerability while maintaining antitumor efficacy [28, 35, 36, 71]. Several studies have shown that a more acceptable toxicity profile, in terms of decreased rates of hand-foot syndrome and stomatitis/mucositis, can be obtained with a PLD dose of 40 mg/m^2^ every 28 days compared to the traditional dose of 50 mg/m^2^, with comparable response rates and outcomes [26, 32, 33]. According to the studies published, the optimal dose intensity appears to range from 10 mg/m^2^ to 12.5 mg/m^2^ per week (given at doses of 40–50 mg/m^2^ every 4 weeks) when used as a single-agent therapy.

The results obtained with a single-agent PLD in the subgroup of platinum-resistant patients were the basis for the development of PLD/platinum (cis-, carbo-, oxaliplatin) combinations.

The trials that evaluated the combination regimen of cisplatin or carboplatin with PLD showed an overall response rate ranging from 46 to 68% according to the platinum-free interval. In the Rapoport trial, the overall response rates were about 65% in a population including platinum-sensitive (81%) and partially sensitive patients (52.6%) [38].

Cisplatin combination regimen (PLD at 50 mg/mq dosage, plus cisplatin at 60 mg/mq d.1 q 28 days) was also developed showing a moderate tolerability profile (10% grade 2 neurotoxicity, 18% grade 3/4 anemia, 41% neutropenia, and 9% hand-foot syndrome) [34]. Due to these results, the PLD/carboplatin combination was considered more manageable due to the lower neurotoxicity [37–39, 72–74].

In two phase I-II trials PLD has been associated with carboplatin AUC 5-6 in sensitive or partially sensitive (>50%) ovarian or other gynecological cancer patients.In both studies, data of ORR (62 and 68%, resp.), PFS (9.2 and 11.6 months), and median overall survival (OS 23.4 and 32 months) substantially overlap [37, 39].

Based on toxicity results, the authors recommended a PLD dose of 40 mg/m^2^ when given in combination with carboplatin AUC 5, both drugs administered on a 4-week schedule in epithelial ovarian or endometrial carcinoma. 

Gemcitabine is another drug studied in combination with PLD. In several trials (PLD 30 mg/m^2^-gemcitabine 1000 mg/m^2^ days 1–8 every 21 days) this combination has been associated with overall response rates of about 30–35% in the overall population (21–25% in platinum-resistant and 50–53% in platinum-sensitive diseases), with an acceptable toxicity profile. Myelosuppression was the most common toxicity and was found in 35% of patients [41, 42].

Combinations of PLD with oxaliplatin (OXA) have been also reported, with response rates that appear in the range of those reported with PLD/carboplatin. In these trials a very acceptable rate of stomatitis/mucositis and hand-foot syndrome has been shown, likely due to the use of the PLD at the dosage of 30 mg/m^2^, every 21 or 28 days.

Nicoletto et al. [40] published a trial of pegylated liposomal doxorubicin, dosed between 30 and 35 mg/m^2^ with oxaliplatin at 70 mg/m^2^ every 28 days. The overall response rate was 54% with a median survival of 22.5 months. When evaluated according to platinum sensitivity, there was a response rate of 66.7% among the 29 platinum-sensitive patients and of 28.6% in the 14 platinum-resistant patients. There were 5 (12%) grade 3 or 4 toxicities and only 3 patients (7%) required dose reduction. Neutropenia was the treatment limiting toxicity.

Some phase II studies explored the efficacy of PLD associated with topotecan (TPT) [43], as well as paclitaxel (PTX) [44], vinorelbine (VNR) [45], and ifosfamide (IFO) [46]. Overall, response rates of about 28% to 37% with a median PFS of 5.5 to 7.5 months were found, figures which are quite comparable to those reported with other nonplatinum combinations. The association with weekly paclitaxel was well tolerated, as was the PLD/VNR combination [45]. In contrast, PLD/TPT, even if tested at different doses of the two drugs, was characterized by an unacceptable rate of severe anemia (48%), leukopenia (70%), and thrombocytopenia (44%) [43].

### 3.2. PLD Single-Agent Phase III Randomized Trials

Table 2 summarizes the results from randomized trials using PLD alone or in combination in phase III studies [47–52].

In the first trial [48], Gordon randomized 474 ovarian cancer patients at first recurrence (stratified by PFI) to PLD (50 mg/m^2^ every 4 weeks) or topotecan (1.5 mg/m^2^/day for 5 consecutive days every 3 weeks). In platinum-resistant disease (*n* = 255) no significant difference was seen in response rate, PFS, or OS between the two treatment arms, while in platinum-sensitive patients (*n* = 219), median PFS and OS were significantly prolonged in PLD-treated patients compared to TPT-treated patients (*P *value = 0.037 and *P *value = 0.008, resp.). More mature survival analysis confirmed the long-term advantage for platinum-sensitive patients receiving PLD versus TPT (median OS = 27 months versus 17.5 months, hazard ratio (HR) = 1.432, *P *value = 0.017) [49]. Moreover, for partially platinum-sensitive disease (*n* = 122), the HR favored PLD versus TPT (HR = 1.58, *P *value = 0.021). About the tolerability profile, grade 3/4 haematological toxicity occurred more frequently and more severely in TPT compared to PLD; in particular, severe neutropenia was documented in 77% of TPT–treated patients versus 12% of PLD-treated patients (*P* < 0.001), and thrombocytopenia was found in 34% of TPT versus 1% of PLD cases (*P* < 0.001). No case of severe HFS was documented in the TPT arm while it was registered in 23% of PLD-treated patients (*P* < 0.001) with no difference in quality of life perceived by the patient. 

In a second randomized trial conducted by O'Byrne et al. [47], 214 patients (not defined according to platinum sensitivity) were randomized to receive either PLD (50 mg/m^2^ every 4 weeks) or paclitaxel (175 mg/m^2^ every 3 weeks). A preliminary analysis of the data showed that there were no significant differences in response rates, PFS, OS, or rate of adverse events. The study was suspended due to poor accrual, as paclitaxel became incorporated into first-line therapy, so no definitive analysis was carried out.

Several additional phase III trials have been reported, which directly compared single-agent PLD to other single agents (paclitaxel, gemcitabine) in platinum-resistant and partially platinum-sensitive (platinum-free interval 6–12 months) ovarian cancer patients [47, 50, 51]. While side-effect profiles of the agents often differed substantially, these studies essentially revealed the therapeutic equivalence for these agents in this difficult clinical setting.

Two phase III trials compared PLD with gemcitabine in recurrent platinum-resistant or partially sensitive ovarian cancer patients [50, 51].

In both trials there was no difference in the response rates and median PFS between the two treatment arms. The median OS in the MITO3 trial was greater in the PLD arm (14 versus 12.7 months, respectively, *P *value = 0.048). With the limits inherent in the small sample series, the survival advantage reported with PLD over GEM was maintained in the subgroup of partially sensitive patients (*P *value = 0.016).

Based on these results PLD at 40 mg/m^2^ seems to offer the most favourable toxicity profile, which is likely to sustain the achievement of better quality of life (QoL) scores (at least in comparison to GEM) and was adopted as a standard worldwide [50].

Other phase III trials have explored the combination of PLD with other nonplatinum agents. Among the most intriguing novel drugs, trabectedin (TRAB) (ET743; Yondelis) has become relevant for treatment of sarcomas and other solid tumors for its unique mechanism of action, in that, unlike most other agents, it binds to the minor groove of DNA thus affecting a variety of transcription factors, cell proliferation, and the nucleotide excision repair system and inhibits the MDR-1 gene coding for the protein responsible for chemoresistance [75–77].

Based on safety and efficacy results from phase-I/II studies, a phase-III trial (OVA-301, NCT00113607) has been performed to compare PLD (50 mg/m^2^ every 28 days) with the combination PLD (30 mg/m^2^) and TRAB (1.1 mg/m^2^ every 21 days) in second-line relapsed ovarian cancer patients, unsuitable for platinum therapy, stratified according to the PFI (PFI < 6 months versus PFI > 6 months). After a median followup of 47.4 months, in the whole series, the response rate was significantly higher in the combination compared to the PLD arm, as was also median PFS (HR = 0.79, *P *value = 0.019) [52].

However, in platinum-resistant cases (*n* = 242) no statistically significant difference was observed with the doublet in terms of response rate (13.4% versus 12.2%, resp.) and PFS, while a clear advantage favouring the combination compared to single-agent PLD was evident in platinum-sensitive disease (RR 35.3% versus 22.6%, *P* = 0.0042; median PFS 9.2 months versus 7.5 months; HR = 0.73, *P* = 0.017) and partially sensitive disease with median PFS of 7.4 months versus 5.5 months in PLD/TRAB versus PLD arm (HR = 0.65, *P* = 0.0152). An unplanned hypothesis-generating analysis adjusting for the PFI imbalance and other prognostic factors suggested an improvement in OS associated with the trabectedin/PLD arm (HR = 0.82; 95% CI: 0.69–0.98; *P* = 0.0285). In another unplanned exploratory analysis, the subset of patients with a PFI of 6–12 months had the largest difference in OS (HR = 0.64; 95% CI: 0.47–0.86; *P* = 0.0027). Data showed a longer time to the following platinum therapy, and this imbalance in platinum-free interval was suggested as a possible cause of the increased OS [78]. Thus, these data suggest that the treatment with an effective nonplatinum combination may artificially prolong the platinum-free interval giving more chance of activity to further platinum therapy. This hypothesis will be investigated in a phase III trial, called INNOVATYION.

As expected the combination regimen of TRAB/PLD has been associated to a greater haematological toxicity (grade 3/4 anaemia 14%, neutropenia and thrombocytopenia 63%). Among other toxicities, short-lived grade 3/4 hypertransaminasemia (38%) and HFS were documented in 4% of the PLD/TRAB arm compared to 20% in the PLD alone arm [79]. In September 2009, based on these results, which support the PLD/TRAB combination as the most effective nonplatinum-based combination in platinum-sensitive disease, the PLD (30 mg/m^2^) and TRAB (1.1 mg/m^2^) association every 3 weeks has been approved by the EMA for treatment of patients with relapsed platinum-sensitive OvCa [80]. 

Based on the phase-II trials in platinum-sensitive OvCa the combination of PLD/carboplatin has been explored in phase-III trials [53]. Markman et al. compared single-agent carboplatin to its combination with PLD in recurrent ovarian cancer, showing a statistically significant improvement of PFS with carboplatin/PLD, without an overall survival benefit. Interestingly, for unknown reasons, the association drastically reduced the rate of hypersensitivity reactions compared to carboplatin alone (9% versus 0%, *P* = 0.0008) [53]. Later on the results of the CALYPSO trial have been reported [81, 82]. This international open-label phase-III trial compared carboplatin PLD (CD) with carboplatin-paclitaxel (CP) in patients with platinum-sensitive recurrent ovarian cancer (ROC). A total of 976 recurrent patients relapsing >6 months after first- or second-line therapy were randomized to receive CD or CP for six cycles. 

Designed as a noninferiority trial, CALYPSO demonstrated that the combination of CD was not only noninferior to CP in terms of PFS, but indeed it was more effective (HR = 0.82, *P* = 0.005) in patients with platinum-sensitive recurrent ovarian cancer. Nevertheless, with a median followup of 49 months, no statistically significant difference in OS was observed (hazard ratio = 0.99 (95% confidence interval 0.85, 1.16) log⁡ rank⁡ *P* = 0.94), with median survival times of 30.7 (CD) and 33.0 months (CP). Treatment-related serious adverse events were more frequent in the CP arm (76 patients (30%) versus 44 patients (18%)), while the CD treatment was associated with more grade 3/4 thrombocytopenia and more grade ≥2 mucositis and PPE. Interestingly, even in this trial as in other phase-II studies there was a lower incidence of allergic reactions, alopecia, neuropathy, and arthralgia/myalgia. PLD/carboplatin represents a valid alternative to other platinum-based regimens in recurrent platinum-sensitive OvCa especially for patients whose QoL is recognized to be heavily compromised by alopecia or who had experienced or had not yet been rescued from taxane-induced neurotoxicity [81, 82].

Attempts to include PLD in a front-line treatment have also been made; in particular, with the aim of improving standard chemotherapy with carboplatin-paclitaxel, doublet or triplet combinations including PLD have been investigated based also on the very favourable and not overlapping toxicity profile. The potential efficacy of triplets and sequential doublets (with TPT, PLD, and gemcitabine) has been investigated in the GOG182/ICON5 trial that enrolled 4312 stage-III/IV patients who were randomized to 5-arm first-line chemotherapy regimens and sequences, with disappointing results. There was no PFS or OS advantage with sequential doublets or with triplets compared with the control arm. In this trial, PLD at a dosage of 30 mg/m^2^ was added to carboplatin and paclitaxel at full dose every other cycle [83].

In the front-line setting, MITO-2 was the first trial investigating the PLD/carboplatin (30 mg/m^2^, AUC = 5, every 21 days) combination compared to the standard treatment; this trial was designed to show a superiority for the carboplatin/PLD combination. Unfortunately, there were no statistically significant differences in either PFS or overall survival between the treatment arms with median PFS times of 19.0 months versus 16.8 months (HR, 0.95; 95% CI, 0.81 to 1.13; *P* = 0.58) and median overall survival times of about 61 and 53 months with carboplatin/PLD and carboplatin-paclitaxel, respectively, (HR, 0.89; 95% CI, 0.72 to 1.12; *P* = 0.32) [84]. Carboplatin/PLD also produced a similar response rate but different toxicities (less neurotoxicity and alopecia but more hematologic adverse effects).

Although the proposed combination has failed to undermine the primacy of the standard carboplatin-paclitaxel, given the observed confidence intervals and the different toxicity, carboplatin/PLD could be considered an alternative to standard first-line therapy, particularly in patients that cannot receive paclitaxel.

## 4. PLD in Epithelial Ovarian Cancer: Future Directions

Based on the excellent results obtained by the PLD alone or in combination with platinum as well as nonplatinum agents in almost all clinical settings of ovarian cancer, early phase trials have begun to explore the potential of adding PLD to a variety of alternative drugs, including bevacizumab (BEV) and other “targeted agents” in the management of epithelial ovarian cancer (Table 3). 

Despite the encouraging results obtained in ovarian cancer, the combination of PLD with bevacizumab was introduced with caution because of the potential mechanism of interference. We know that the increased vascular permeability known as “EPR effect” greatly enhances liposome deposition in tumors enabling the increase of intratumoral delivering and concentration of PLD. Normalization of the vasculature induced by bevacizumab has been hypothesized to interfere with liposomal tumour entry, but a concomitant reduction in tumour interstitial pressure, on the other hand, could improve PLD delivery. In a trial conducted by Muggia et al. the pharmacokinetic of PLD alone or in combination with bevacizumab was investigated in order to evaluate the postulated interferences. Trial results show an increased PLD T 3/4, C7d/Cmax, and PLD levels at day 21 after bevacizumab introduction, probably reflecting a greater delivery of PLD to tumours [55]. Preliminary results from a phase II study with the PLD/BEV combination in platinum-resistant patients have been presented by the same authors. The study was conducted on 48 patients. PLD (30 mg/m^2^ every 21 days) was administered alone at the first cycle, and then with BEV (15 mg/kg every 21 days) for the following 6 cycles or until progression [85].

This proof-of-concept study was the first to report the efficacy and the tolerability of the combination of PLD and bevacizumab in the treatment of recurrent ovarian cancer. The ORR observed in this trial was 72.2% (95% CI: 58.4, 83.5). The safety profile was consistent with the known toxicities of these agents with no sign of overlapping toxicities nor any reports of cumulative-dose cardiotoxicity. 

Following these data a large phase III randomized study (AURELIA) in platinum-resistant setting assessed the efficacy of bevacizumab (10 mg/kg every 2 weeks or 15 mg/kg every 3 weeks) combined to either dose-dense paclitaxel (80 mg/m^2^ weekly), topotecan (4 mg/m^2^ on days 1, 8, and 15 of each 4-week cycle or 1.25 mg/m^2^ on days 1 through 5 of each 3-week cycle), or pegylated liposomal doxorubicin (40 mg/m^2^ every 4 weeks). After a median followup (after 301 PFS events) of 13.5 months, the overall response rates (ORR) were 30.9% in the bevacizumab combination arm compared to 12.6% of chemotherapy alone (HR 0.48; CI 95%). In platinum-resistant OC, bevacizumab combined to chemotherapy provided a statistically significant and clinically meaningful improvement in PFS and ORR compared to chemotherapy alone with an acceptable safety profile also due to strict inclusion criteria that minimized the incidence of BEV adverse events. This is the first phase-III trial in platinum-resistant ovarian cancer that shows a clear benefit with a targeted agent combination regimen associated to an improved outcome compared to monotherapy [56]. Taken overall these data suggest that there is no pharmacologic disadvantage of the combination of PLD with bevacizumab.

In platinum-sensitive ovarian cancer relapse bevacizumab has been associated with carboplatin/PLD regimen in another phase-II trial with promising results. Among the 54 patients enrolled, the ORR was 72.2% (95% CI: 58.4, 83.5), the median duration of response was 11.9 months, and median TTP was 13.9 months (95% CI: 11.4, 16.0). The safety profile was consistent with the known toxicities of these agents, making this association a potential treatment option for platinum-sensitive ovarian cancer patients [57].

PLD is also under investigation with other antiangiogenetic drugs. A phase-III ongoing trial (TRINOVA 2 study) compares PLD to PLD in association with AMG386, an angiopoietin inhibitor [59]. 

Panitumumab is a fully human monoclonal antibody specific to the epidermal growth factor receptor (EGFR). No previous studies have evaluated the effect of panitumumab in ovarian cancer (OC) based on KRAS mutation status. The main purpose of the PaLiDo study, a phase-II nonrandomized multicenter trial presented at ASCO 2012 [58], was to investigate the response rate in platinum-resistant, KRAS wild-type OC patients treated with PLD and panitumumab. Patients with relapsed and pretreated (no more than two lines) ovarian cancer were treated with panitumumab (6 mg/kg days 1 and 15) and with PLD (40 mg/m² day 1) every 4 weeks. Progression-free and overall survival in the intention-to-treat population (N 543) was 2.7 months (2.5–3.2 months, 95% CI) and 8.1 months (5.6–11.7 months, 95% CI), respectively, with a considerable skin toxicity, grade 3 in about 40% of patients.

Other phase-I trials evaluated PLD in combination with the mTOR inhibitor temsirolimus [60] and with the folate receptor ligand farletuzumab [86] (humanized monoclonal antibody that binds to folate receptor-*α*, a target which is largely absent in normal epithelium and overexpressed in EOC) showing feasibility and activity. 

Data regarding combinations are very preliminary, but, at least with antiangiogenetic drugs, the combination seems tolerable and active.

Another field of development is that of the patients with BRCA mutation. BRCA1- or BRCA2-mutated ovarian cancer patients are defective of the mechanisms of DNA repairing. This determines an improved chemosensitivity to some DNA-damaging agents [87]. PLD that leads to DNA damage by inhibiting topoisomerase II may prove to be more effective in these patients [88]. In a recent study from Kaye et al. [89], the PARP inhibitor olaparib was compared with PLD in BRCA-mutated patients. The study showed significant single-agent olaparib activity while PFS was not significantly improved compared to PLD. Interestingly, this negative result was hypothesis generating based on the unexpected high PFS found in the control PLD arm. In fact, the 7.1-month PFS observed in this study with PLD was significantly higher than that expected for this drug in the general population. These results are in accordance with retrospective data published by Adams and colleagues on Gynecologic Oncology in 2011 confirming the higher activity of PLD in BRCA-mutated ovarian cancer patients. Although all these data are very preliminary, it seems that PLD may have a special role in patients with BRCA mutation or BRCAness profile [90]. In the same direction are the results of a multicentre retrospective study in relapsed ovarian patients, BRCA mutation carriers, treated with PLD, where Safra et al. showed an improved outcome in terms of median time to treatment failure (15.8 months versus 8.1 months in nonhereditary OC) and overall survival (56.8 months versus 22.6 months) [91]. 

## 5. Conclusions

PLD plays an important role in the management of ovarian cancer. It represents the standard therapy in platinum-resistant recurrence and one of the standard options in platinum-sensitive patients. Between the combination regimes, due to the results of efficacy achieved in phase-II and -III trials and considering the favorable safety profile, carboplatin/PLD represents a valid alternative in both first-line (in patients that cannot receive paclitaxel) and recurrent ovarian cancer compared to actual standard options.

Combination with nonplatinum agents (trabectedin), and antiangiogenetic drugs (bevacizumab) represents an alternative treatment option in the recurrent setting, associated in certain cases with remarkable toxicity. New target therapy is under evaluation in combination with PLD.

## Figures and Tables

**Table 1:** Phase-II studies with pegylated liposomal doxorubicin (PLD) as a single agent or in combination regimens.

Author	Dose/schedule	Clinical setting PFI (mts)	No. pts	RR (%)	PFS (median) (mts)
Muggia et al. [25]	50 mg/m^2^, q21	≤6	35	25.7	5.7
Gordon et al. [18]	50 mg/m^2^, q21	ALL	89	16.8	4.8
Rose et al. [26]	50 mg/m^2^, q28	≤6	37	13.5	4.0
	40 mg/m^2^, q28			7.7	4.0
Katsumata et al. [28]	50 mg/m^2^, q28	≤6	63	20.9	5.6
Markman et al. [31]	40 mg/m^2^, q28	≤6	44	9.1	—
		ALL		13.5	7.2
Lorusso et al. [35]	35 mg/m^2^, q21	≤6	17	18.9	—
		≥6	20	10.0	—
Sehouli et al. [36]	20 mg/m^2^, q15	ALL	64	10.9	4.3
Du Bois et al. [37]	PLD (40 mg/m^2^) d1 CBDCA (AUC 6) d1, q28	≥6	67	68	11.6
Rapoport et al. [38]	PLD (50 mg/m^2^) d1 CBDCA (AUC 5) d1, q28	ALL 7–12	40 19	67.5 52.6	11.9 9.7
Ferrero et al. [39]	PLD (30 mg/m^2^) d1 CBDCA (AUC 5) d1, q28	ALL 7–12 ≥12	96 43 53	62.5 — —	9.4 7.9 11.4
Nicoletto et al. [40]	PLD (30 mg/m^2^) d1 OXA (70 mg/m^2^) d1, q28	≤6 ≥6	14 29	28.6 66.7	5.9 9.9
D'Agostino et al. [41]	PLD (30 mg/m^2^), d1 GEM (1000 mg/m^2^), d1, 8 q21	≤12 ≥12	36 31	25.0 45.2	— —
Ferrandina et al. [42]	PLD (30 mg/m^2^), d1 GEM (1000 mg/m^2^), d1, 8 q21	RES ≥12	66 45	21.6 53.7	5 8.7
Verhaar- Langereis et al. [43]	PLD (30 mg/m^2^) d1/TPT (1.0 mg/m^2^) d1–5 q21 and PLD (40 mg/m^2^), d1 TPT (0.75 mg/m^2^), d1–5 q21	≤12	27	28.0	7.5
Campos et al. [44]	PLD (30 mg/m^2^), d1, q21 PTX (70 mg/m^2^), weekly	ALL ≤12 ≥12	37 24 13	29.0 17.0 54.0	—
Katsaros et al. [45]	PLD (30 mg/m^2^), d1 vinorelbine (30 mg/m^2^), d1, q21	ALL	30	37.0	5.5
Joly et al. [46]	PLD (40 mg/m^2^), d1 ifosfamide (1700 mg/m^2^), d1–3 q28	ALL RES SEN	98 57 41	28.0 19.0 41.0	—

PFS: progression-free survival; RR: response rate; RES: platinum-resistant recurrent disease (platinum sensitivity according to the cutoff of 12-month platinum-free interval); SEN: platinum-sensitive recurrent disease; q: every; d: day; CDDP: cisplatin; CBDCA: carboplatin; PFI: platinum-free interval; GEM: gemcitabine; PTX: paclitaxel; TPT: topotecan; OS: overall survival.

**Table 2:** Phase-III studies with pegylated liposomal doxorubicin (PLD) as a single agent or in combination regimens.

Author	Dose/schedule	Clinical setting PFI (mts)	No. pts	RR (%)	PFS (median) (mts)	OS
O'Byrne et al. [47]	PLD (50 mg/m^2^) q28 versus PTX (175 mg/m^2^) q21		214			
		REC	107	17.8	5.4	11.4
			107	22.4	6.0	14.0
Gordon et al. [48, 49]	PLD (50 mg/m^2^) d1, q28 versus TPT (1.5 mg/m^2^) d1–5 q21	RES	255 130 125	12.3 6.5	2.3 3.4	8.9 10.3
Mutch et al. [50]	PLD (50 mg/m^2^) d1, q28 versus GEM (1,000 mg/m^2^) d1, 8, q21	RES	195 96 99	8.3 6.1	3.6 3.1	12.7 13.5
Ferrandina et al. [51]	PLD (40 mg/m2) q28 versus GEM (1,000 mg/m^2^) d1, 8, 15 q28	RES	153 76 77	16 29	4.0 5.0	14 12.7*
Monk et al. [52] OVA-301	TRAB (1.1 mg/m^2^) d1, q21 versus PLD (50 mg/m^2^) q28	ALL	672	28.0* 19.0	7.3* 5.9	20.5 19.4
	PLD (30 mg/m^2^) d1 TRAB (1.1 mg/m^2^) d1, q21 versus PLD (50 mg/m^2^) q28	SEN	430			
			335 337	35* 23	9.2* 7.5	— —
Markman et al. [53] SWOG SO200	PLD (30 mg/m^2^) d1/CBDCA (AUC 5) d1, q28 versus CBDCA (AUC 5) d1, q28	SEN 6–24 mts	31 30	59* 28	12* 8	31 18
Pujade-Lauraine et al. [54] CALYPSO	PLD (30 mg/m^2^) d1 JM8 (AUC 5) d1, q28 versus PTX (175 mg/m^2^) d1 JM8 (AUC 5) d1, q21	SEN >6 mts	467 509	— —	11.3* 9.4	— —

GEM: gemcitabine; OS: overall survival; PFS: progression-free survival; PTX: paclitaxel; REC: not otherwise specified recurrent disease; RES: platinum-resistant recurrent disease; RR: response rate; SEN: platinum-sensitive recurrent disease; TRAB: trabectedin; q: every; d: day. *Statistically significant.

**Table 3:** Phase-I-II-III studies with pegylated liposomal doxorubicin (PLD) in combination with target agents.

Author	Dose/schedule	Clinical setting PFI (mts)	No. pts	RR (%)	PFS (median) (mts)
Muggia et al. [55]	PLD 30 mg/m and BEV 15 mg/kg on cycles 2–7 (with option to continue)	≤6	48	Ongoing	Ongoing

Pujade-Lauraine et al. [56]	*Arm1* PTX (80 mg/m^2^) d1, 8, 15 and 22 q28 or TPT (4 mg/m^2^) d1, 8, 15 q28 or PLD (40 mg/m^2^) d1 q28	≤6	166	12.6	3.4
	*Arm2* BEV 10 m/kg d1 q15 or 15 mg/kg d1 q21, PTX (80 mg/m^2^) d1, 8, 15 and 22 q28 or TPT (4 mg/m^2^) d1, 8, 15 q28 or PLD (40 mg/m^2^) d1 q28		135	30.9	6.7

Del Carmen et al. [57]	PLD (30 mg/m^2^) d1 q28, CBDCA (AUC5) d1 q28, Beva 10 mg/kg d1 q14	≥6	54	72.2	13.9

Steffensen et al. [58]	PAN 6 mg/kg d1, 15 q28/PLD 40 mg/m² day 1 q28	≤6	46	24.3	2.7–8.1

TRINOVA 2 [59] http://clinicaltrials.gov/show/NCT01281254	*Arm 1* PLD 50 mg/m² d1 q28 and blinded AMG 386 15 mg/kg qw *Arm 2* PLD 50 mg/m² d1 q28 and blinded AMG 386/placebo qw	≤12	Ongoing	Ongoing	Ongoing

Boers-Sonderen et al. [60]	T 15–20 mg/m²/PLD 20–40 mg/mq	ALL	20	3PR 9SD	4.9

PFS: progression-free survival; PTX: paclitaxel; TPT: topotecan; T: temsirolimus; PAN: panitumumab; BEV: bevacizumab; RR: response rate; SEN: platinum-sensitive recurrentdisease; TRAB: trabectedin; q: every; d: day. *Statistically significant.
